# A wearable deep brain stimulation system for behavioral studies in rodents

**DOI:** 10.3389/fnins.2025.1707636

**Published:** 2026-01-05

**Authors:** Yuchen Shen, Dechun Zhao, You Lv, Yanghuazi Liu, Yi Chen, Yuan Zhang, Siyu Chen

**Affiliations:** The School of Life Health Information Science and Engineering, Chongqing University of Posts and Telecommunications, Chongqing, China

**Keywords:** deep brain stimulation, behavioral, stimulator, rodents, embedded system

## Abstract

**Introduction:**

Deep brain stimulation (DBS) has been demonstrated to improve motor function by modulating brain circuits. However, the precise mechanism of action remains unclear. Existing DBS devices often lack suitability for rodent models, which are essential for exploring these mechanisms. The objective of this study is to design a wearable DBS device that enables more effective rodent behavior research.

**Methods:**

We developed a stimulator specifically designed for rodent behavioral research. The device features several key advantages, including low weight (5 g excluding battery), compact size (30 mm × 38 mm), and extended battery life (≥300 hours). Additionally, it integrates low-power Bluetooth, allowing for real-time wireless adjustment of stimulation parameters, such as frequency (80–150 Hz), pulse width (40–340 μs), and amplitude (2.3–3 V).

**Results:**

The stimulating effect of this device has been verified in a model of mild Parkinson’s disease (mild Parkinsonism) in rats, indicating that the device is effective in regulating the motor functions of rodents with Parkinson’s disease-like conditions.

**Discussion:**

This wearable DBS device shows broad applicability in behavior-based studies and offers a valuable tool for investigating the mechanisms of various brain nuclei in rodent models. It holds significant potential for enhancing the exploration of DBS effects in clinical and experimental settings.

## 1 Introduction

Deep brain stimulation (DBS), an electro-physiological neuromodulation technique, has emerged as a revolutionary therapeutic approach (Xu et al., 2025). With the continuous advancement of technology, its clinical application has expanded beyond Parkinson’s disease (PD) and essential tremor to encompass a range of neuropsychiatric disorders, such as dystonia, stroke, obsessive-compulsive disorder and epilepsy (Kupsch et al., 2006; Deuschl et al., 2011; Menchón et al., 2021; Ryvlin and Jehi, 2022; Baker et al., 2023; Shin et al., 2023; Hoang et al., 2025; Xu et al., 2025). The subthalamic nucleus (STN), a key node in the basal ganglia circuit involved in motor control, is one of the most common DBS targets for treating motor symptoms in PD, including bradykinesia and tremor (Rodriguez-Oroz et al., 2005; Xu et al., 2025). In clinical practice, high-frequency (>100 Hz) DBS (HF-DBS) has become the standard treatment for refractory PD (Benabid, 2003; Benabid et al., 2009; Bahadori-Jahromi et al., 2022; Baker et al., 2023). However, improper selection of DBS stimulation parameters may lead to side effects or reduce treatment efficacy (Cagnan et al., 2019; Lozano et al., 2019). As a result, there is a growing need for optimization of DBS parameters and PD signal identification, which has driven significant research into DBS tools for PD models, such as rodents (Burton et al., 2021).

To better understand the complex biological mechanisms underlying Parkinson’s disease (PD) and deep brain stimulation (DBS), translational research using biological disease models is essential (Çağnan, 2010; Burton et al., 2021; Grotemeyer et al., 2024). However, in rodent DBS research, existing devices have followed two distinct paths. The wired DBS system has significantly contributed to previous studies, but its limitations are apparent, particularly its susceptibility to interference from the test environment and biological factors (Knorr et al., 2022). On the other hand, the wireless DBS system offers enhanced flexibility and repeatability for research by eliminating the need for a connection between the device and the host computer (Forni et al., 2012; Pinnell et al., 2018; Adams et al., 2019; Alpaugh et al., 2019; Fleischer et al., 2020). Despite its advantages, the wireless DBS system faces challenges related to its size and energy consumption, which can complicate long-term research and validation (Fluri et al., 2017; Pinnell et al., 2018; Fleischer et al., 2020; Knorr et al., 2022).

Recently, the design of neural stimulation devices has shown a diverse trend, particularly in terms of adaptability to various experimental needs and application scenarios. Based on the placement or implantation locations, these devices can be roughly categorized into head-mounted (Fluri et al., 2017; Pinnell et al., 2018), backpack (Even-Chen et al., 2020; Tala et al., 2021; Paul et al., 2025), and subcutaneously implanted types (Forni et al., 2012; Alam et al., 2013; Alpaugh et al., 2019; Fleischer et al., 2020; Plocksties et al., 2021; Grotemeyer et al., 2024; Plocksties et al., 2025). According to the method of signal transmission, they can further be classified into wired and wireless types (Alam et al., 2013; Fluri et al., 2017; Even-Chen et al., 2020; Tala et al., 2021; Grotemeyer et al., 2024). For instance, the head-mounted wireless microstimulator proposed by Fluri et al., designed specifically for stroke rat models, minimizes interference with the natural behavior of the rats due to its compact, flexible, and lightweight design. However, it also has limitations, including restricted adjustable stimulation parameters and relatively highly power consumption (Fluri et al., 2017). A programmable, miniaturized device for water maze testing, proposed by Pinnell et al., innovatively applies 3D printing technology to the device’s housing, thereby extending the application scenarios of existing DBS devices. However, this device is more suitable for acute experiments, and its weak battery life limits its application in long-term research (Pinnell et al., 2018). Paul et al. proposed a wireless brain stimulator for rodent behavioral research, which verified stimulation efficacy using both intracranial and cortical stimulation methods. However, the device only provides open-loop stimulation based on experimental requirements, and its large size and limited battery life present challenges for long-term use (Paul et al., 2025).

In backpack devices, Tala et al. developed a low-cost wireless multichannel DBS system that secures the wireless transmission and stimulation modules on the back of the experimental animal using a harness. This design provides greater flexibility and adjustability, but the system faces a significant issue of insufficient battery life (Tala et al., 2021). Additionally, Even-Chen et al. proposed a backpack device that only features signal acquisition functionality, without integrated stimulation circuitry, thus limiting its scope of application (Even-Chen et al., 2020). Moreover, such devices generally suffer from the common drawback of excessive bulkiness, which adds additional strain on the experimental animals.

Subcutaneously implanted neural stimulators have the advantage of small size and long operational duration, making them suitable for long-term research. However, implantation surgery can lead to infection, increased discomfort for the animals, and added technical difficulty in battery replacement (Fleischer et al., 2020; Grotemeyer et al., 2024). For example, Alpaugh et al. developed an implanted microprocessor that combines conventional electrical stimulation with 2-photon imaging by adjusting the spatial position and orientation (vertical and horizontal) of the electrodes. While this setup allows for effective deep brain stimulation, it does not allow for rapid adjustments to stimulation parameters after implantation, which limits its flexibility and real-time responsiveness (Alpaugh et al., 2019). Forni et al. introduced a compact and implantable microstimulator, successfully overcoming the limitations of traditional wired systems. Despite its small size and convenience, which provide advantages in certain experiments, it faces limitations in parameter adjustment and does not support closed-loop stimulation (Forni et al., 2012). Furthermore, the software-defined implantable modular platform (STELLA) proposed by Plocksties et al. offers a flexible and programmable stimulation solution that provides strong support for preclinical deep brain stimulation research in rodents. However, once fully encapsulated and implanted, the platform’s internal program cannot be modified, and the stimulation parameters cannot be determined before implantation, which limits its flexibility and adaptability (Plocksties et al., 2021). Hoang et al. proposed a fully wireless, implantable multi-channel neural spike stimulation and recording device for epilepsy. This device addresses the issue of battery life by utilizing a wireless power transfer (WPT) spiral coil and employs multi-channel electrodes for both signal acquisition and stimulation. However, the device’s compact layout leads to interference and noise between circuits, which reduces its performance. Additionally, once implanted, the device does not support firmware updates, significantly increasing the risk for subsequent experiments (Hoang et al., 2025).

In summary, while both wired and wireless DBS systems have unique advantages and application potential, they also exhibit varying degrees of limitations (Grotemeyer et al., 2024). In the long-term behavioral research applicable to rodents, there are still some problems such as too large volume, high power consumption, inflexibly parameter adjustment, and unadjustable after implantation. To meet the multi-objective, single-port, multi-site, and long-duration requirements of rodent clinical DBS research, it is imperative to develop a device that overcomes the deficiencies of existing equipment while optimizing clinical experimental validation. Therefore, in device design, it is necessary to integrate the advantages of wireless technology and balance the key parameters such as flexibility, power consumption, size and operability. The purpose is to ensure the stimulation efficiency, while taking into account the lightweight, low power consumption and high flexibility of the device.

Considering the existing stimulation strategies of DBS devices, it is necessary to design a wireless and wearable rodent DBS device that can overcome the technical limitations of multi-site and multi-target. Based on the above design goals, we propose a software platform for a wearable deep brain stimulation device with user-friendly interactive features for rodent behavioral research.

This device consists of a low-impedance microelectrode and microstimulator system that offers two key advantages.

1) It is a wireless control stimulation system, which can control and change the stimulus parameters through the host computer, and has considerable flexibility.
2) As a wearable device, it has minimal interference to the spontaneous behavior of experimental animals. To demonstrate the feasibility of this system, we describe here a step-by-step surgical implantation protocol with rodent behavioral studies.

This design uses a highly integrated low-power circuit architecture, combined with Bluetooth wireless communication technology, to achieve real-time remote adjustment of stimulation parameters (frequency, pulse width, amplitude). Weighing just 5 grams (excluding batteries), the stimulator is compact (38 mm × 30 mm) and equipped with a rechargeable lithium battery that lasts no less than 300 h, significantly better than most existing devices. In addition, we adopted a biphasic pulse output mode to effectively reduce the risk of tissue damage and improve stimulation safety. We expect that this device will provide a reliable, flexible, and easy to operate tool platform for behavior-based neural mechanism research. Furthermore, we illustrate the application of this system in a rat model of hemi-Parkinsonism’ Disease (hemi-PD), in which a PD-like phenotype is induced by stereotactic injection of the neurotoxin 6-hydroxydopamine (6-OHDA) into the medial forebrain bundle (MFB) of rats.

## 2 Design

### 2.1 Circuit structure and design

**Figure 1:**
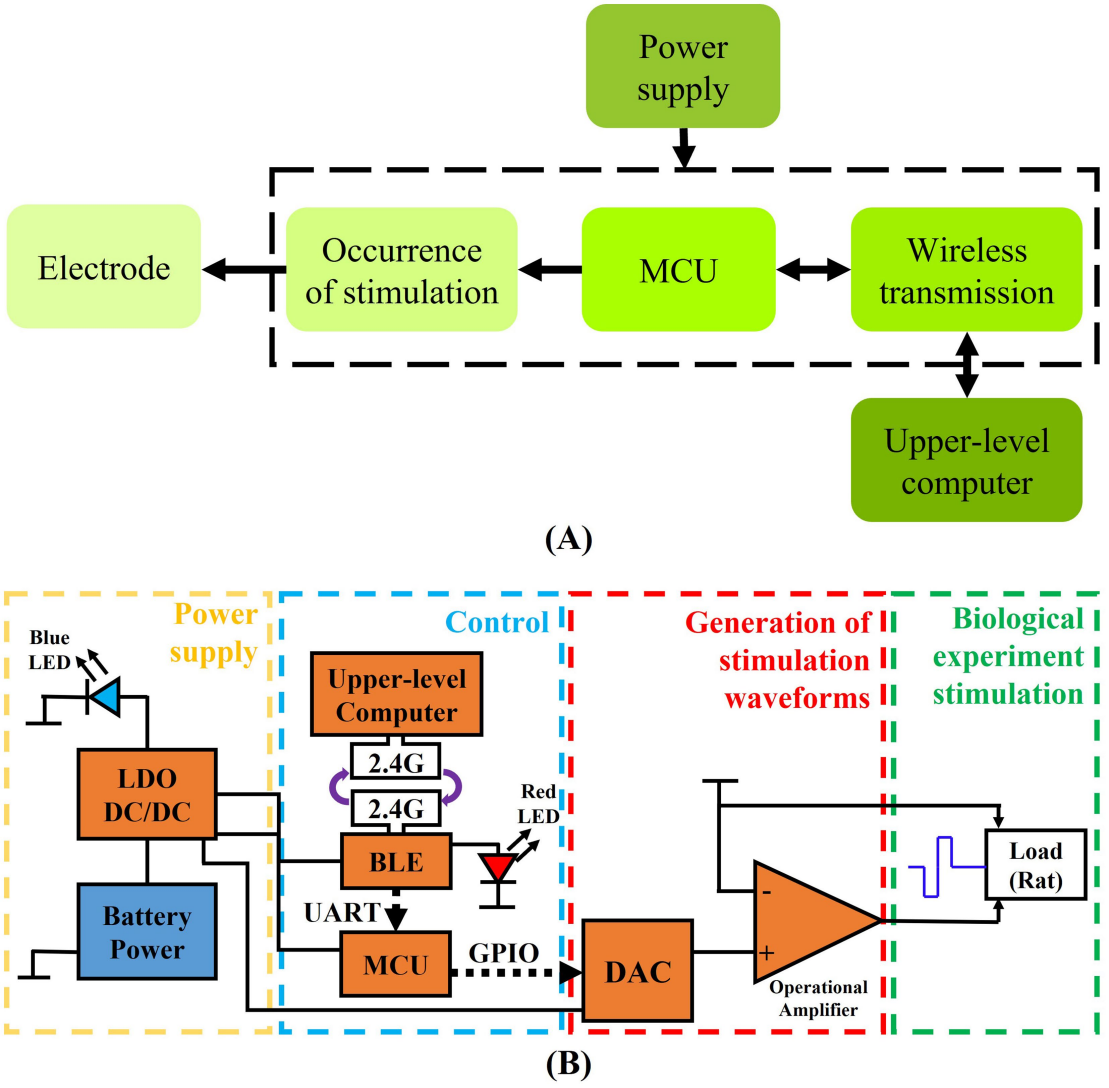
Block diagram of circuit structure and function. **(A)** System framework composed of several modules. **(B)** Block diagram of circuit function between each module.

The system framework and the functional block diagram of the circuit are shown in Figure 1. Among them, MCU of MSP430F5310, DAC8532, OPA703 chips (Texas Instruments, United States) and a commercially available PCB with BLE system (HM-17) are used as the core components.

**Figure 2:**
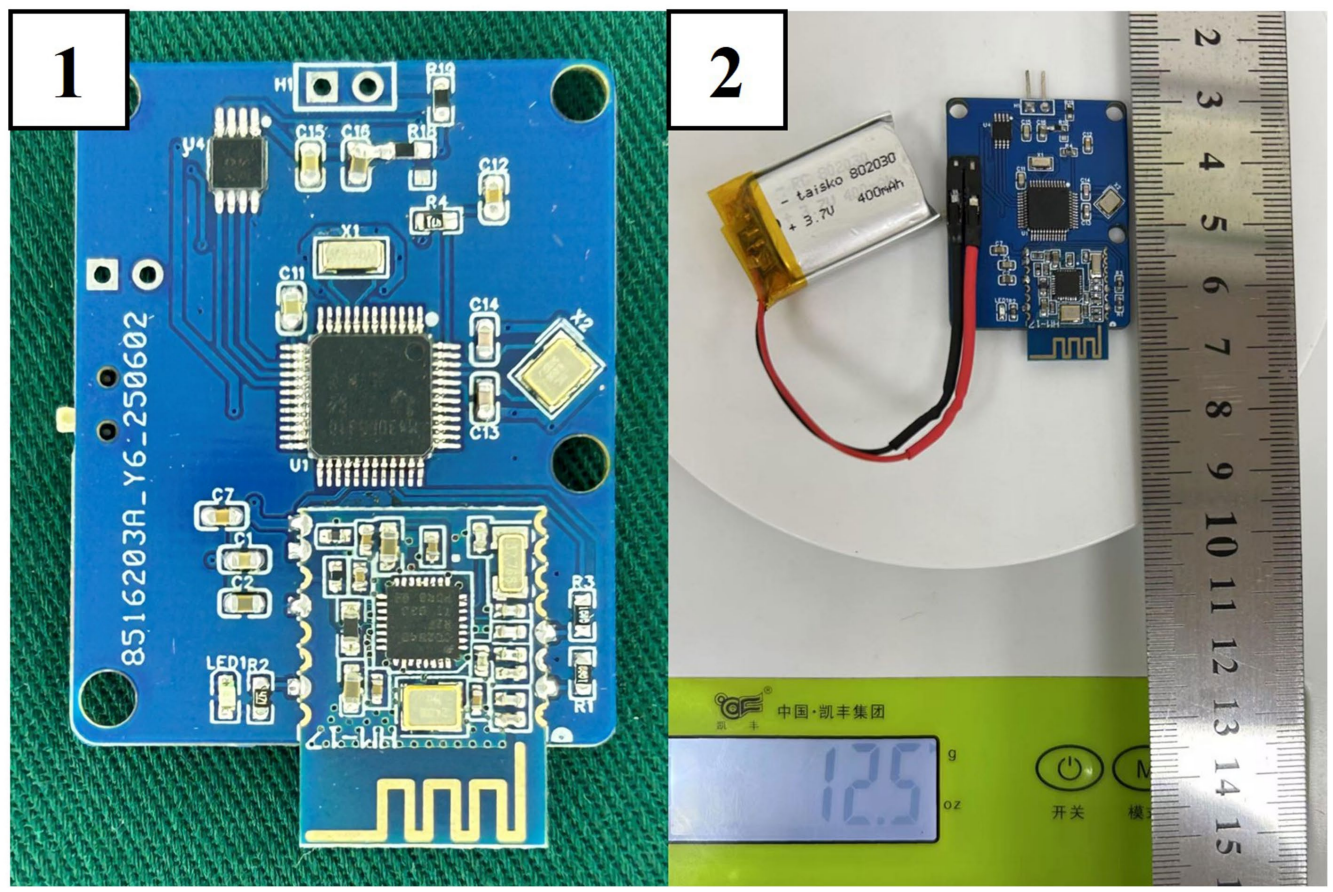
A wearable deep brain stimulation prototype device for behavioral studies in rodents.

The system consists of four functional modules: main control module, voltage stimulation source module, power supply module and wireless transmission module. The PCB size is 38 × 30 mm (length × width) and the weight is 12.5 g. The final product of the wearable deep brain stimulation device for rodent behavioral research is shown in Figure 2.

#### 2.1.1 Main control module

The main control module consists of a 16-bit low-power microcontroller (MSP430F5310) and its associated external circuitry. The chip transmits data to the wireless transmission module through UART communication, and then interacts with the host computer through BLE. Finally, a universal I/O port and an internal timer are used to control the stimulation generation module, and the microstimulator is programmed in IAR for MSP430 software through C language.

#### 2.1.2 Voltage stimulation source module

In this study, an efficient circuit was designed to achieve adjustable voltage output and reduce damage to the target tissue during stimulation. The use of biphasic stimulation pulse can achieve the goal of effectively reducing charge accumulation and improving the safety and effect of stimulation. Specifically, a digital to analog converter (DAC) chip and an operational amplifier (OPA) chip are combined to construct a forward side adder circuit to achieve accurate output of the biphasic pulse. This design helps to ensure current stability during stimulation with minimal tissue intrusion.

**Figure 3:**
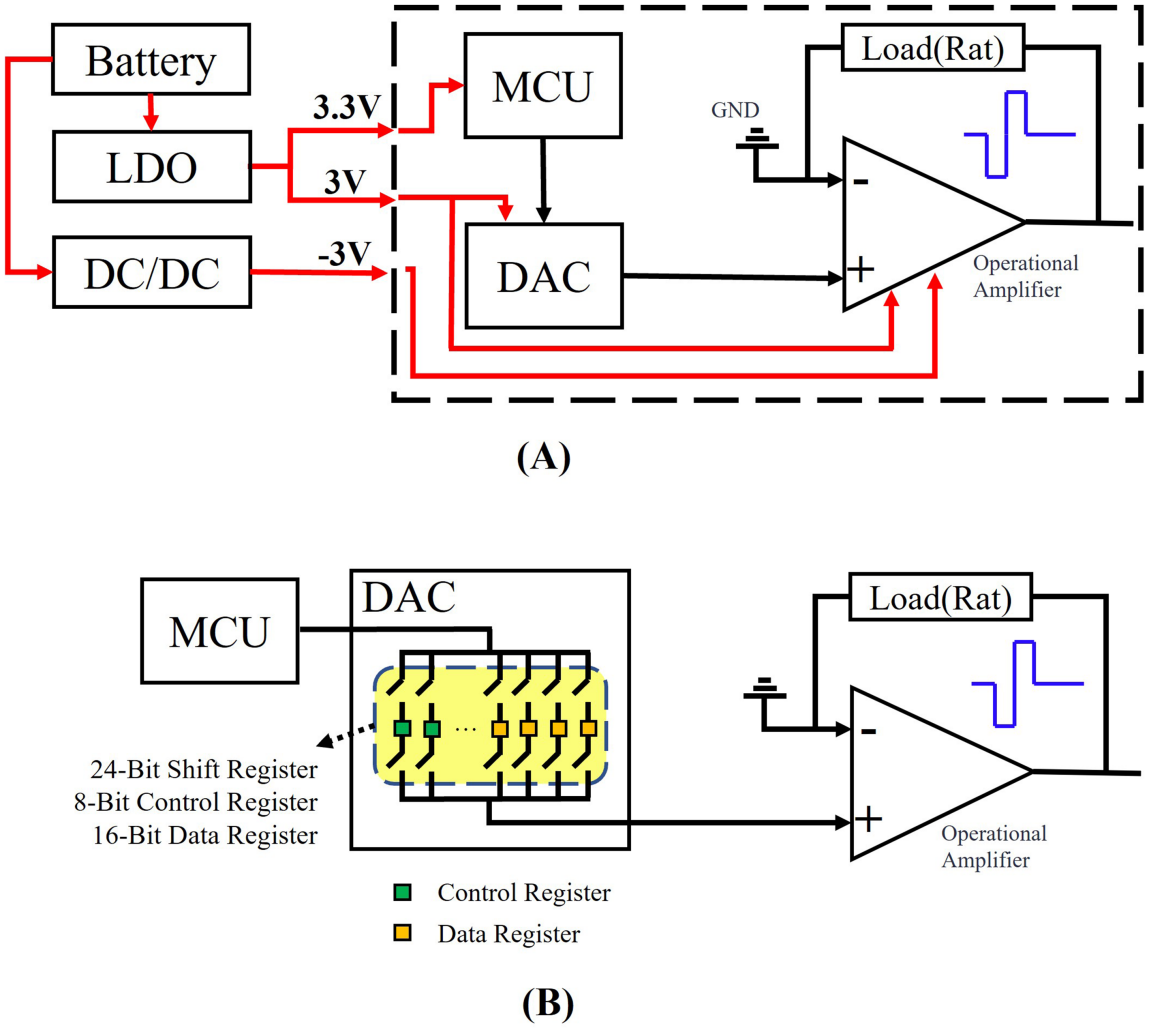
Schematic diagram of voltage stimulation source structure and operation. **(A)** Schematic design of volt-age stimulation source structure. **(B)** The internal structure and working principle of DAC chip are described.

DAC8532 chip realizes analog channel and output mode conversion by writing different data to 8 control bits (DB16-DB23), and uses 16 data bits (DB0-DB15) to control the output voltage amplitude. The high-performance operational amplifier (OPA703) used in the system has low power consumption, significantly extending the device’s battery life. Additionally, this chip features a low offset voltage, ensuring precise signal processing and voltage output. The structure and working principle of the stimulus generation module are shown in Figure 3.

#### 2.1.3 Power supply module

The PSM comprises several key components, a DC/DC converter, and low-dropout voltage regulators (LDOs). The positive LDO composed of TPS73633 (Texas Instruments) provides stable power supply for the microstimulator chip and wireless transmission module. The positive LDO composed of LP5907 (Texas Instruments) and TPS60400 (Texas Instruments) are paired for the power supply of voltage stimulation source to realize the electrostatic discharge (ESD) protection of voltage stimulation source. These LDOs are used to suppress the power ripple generated by the DC/DC converter and ensure a stable power delivery to the modules of the system.

#### 2.1.4 Rated power consumption and low-power sleep mode

In instances where the micro-stimulator was not manually deactivated and remained in standby mode, a low-power sleep mode was automatically engaged to minimize energy consumption. The system consumed power only during Bluetooth communication and stimulation events; otherwise, it entered a sleep state. Upon establishing a Bluetooth connection, the device awakened, resumed operation, and subsequently returned to sleep mode once the connection was terminated.

In order to facilitate experiments on animals with free movement and to ensure that the stimulation duration remains above 300 h, this study employed a commercial 3.7 V (400mAh, 8.2 mm × 20.5 mm × 32 mm) rechargeable lithium battery as the power source, ensuring adequate endurance for the microstimulator system. The researchers can turn the microstimulator on and off via a sliding switch, with its operational status indicated by LED flashing patterns in the peripheral circuitry. Table 1 lists the functional descriptions of the relevant blinking programs. Table 2 summarizes the relevant technical parameters of the microstimulator.

**Table 1:** Flash-code of the LED on the PCB.

LED color	State	Description
Blue	Close	Device OFF
	Constant light	Device ON
Red	Flicker/500 ms	BLE is in standby mode
	Constant light	BLE is in the connected mode

**Table 2:** Technical specification of the stimulator.

Stimulus indicator	Index parameter
Battery life time	≥300 h
Supply voltage (V)	3.7 V DC
Current pulse amplitude (*Vp*)	2.3–3 V
Current frequency range (*f*)	80–150 Hz
Current pulse-width (*tp*)	40–340 μs
Current pulse pattern	Biphasic
Weight (without batteries)	5 g
Batteries Weight	7.5 g
Dimensions (incl. housing)	34 × 42 × 15 mm

### 2.2 Host computer and user interface design

#### 2.2.1 Programmable UI for DBS parameter control

**Figure 4:**
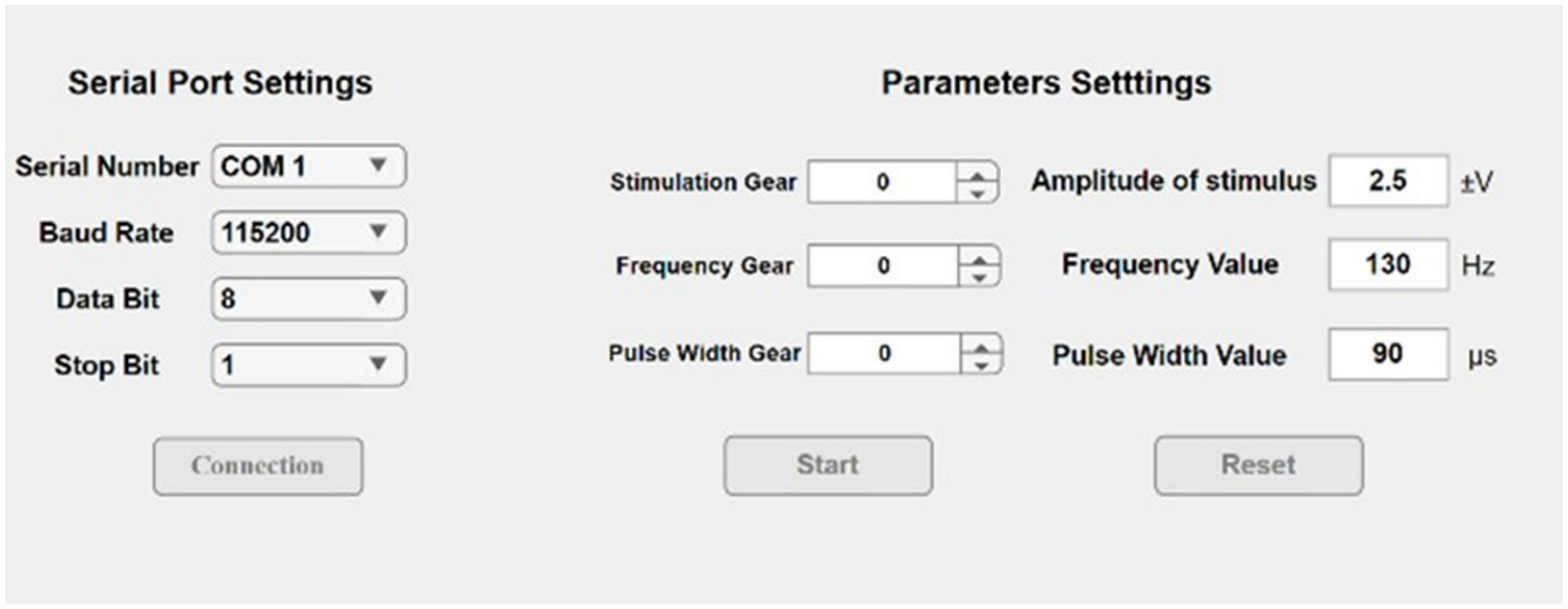
Schematic of the user interface.

In this study, the Matlab2023b app tool (introduced by MathWorks, United States) was used to develop the upper computer software. The interface of the host computer mainly includes two parts: serial port setting and stimulation parameter adjustment. In the serial port setting part, the connection with the main Bluetooth module can be established through the configuration parameters, and the set stimulus parameters can be transmitted to the hardware platform based on the Bluetooth protocol. In the stimulation parameter adjustment part, the Spinner function was used to adjust the output parameter gear, so that the stimulus parameters could be variable and the output waveform of the hardware could be dynamically adjusted. The schematic of the user interface are shown in Figure 4.

#### 2.2.2 DBS stimulation parameter programming

The effect of commercial DBS systems is largely dependent on the parameters of the applied stimulation pulse, including waveform, pulse width, frequency, and amplitude (Magown et al., 2018; Potel et al., 2022; Wang et al., 2024). Compared with different types of stimulation waveforms, the balanced charge of the biphasic pulse output avoids the occurrence of toxic electrochemical reaction products when the single-phase pulse output, so as to ensure that it can prevent irreversible damage to the brain tissue around the electrode contacts. Therefore, more mature clinical DBS will adopt a safer biphasic stimulation pulse mode. For the stimulation frequency, high frequency (>100 Hz) stimulation waveform is the gold standard for the treatment of clinical PD movement disorders, but its long-term continuous stimulation can also lead to attention and cognitive impairment problems.

**Figure 5:**
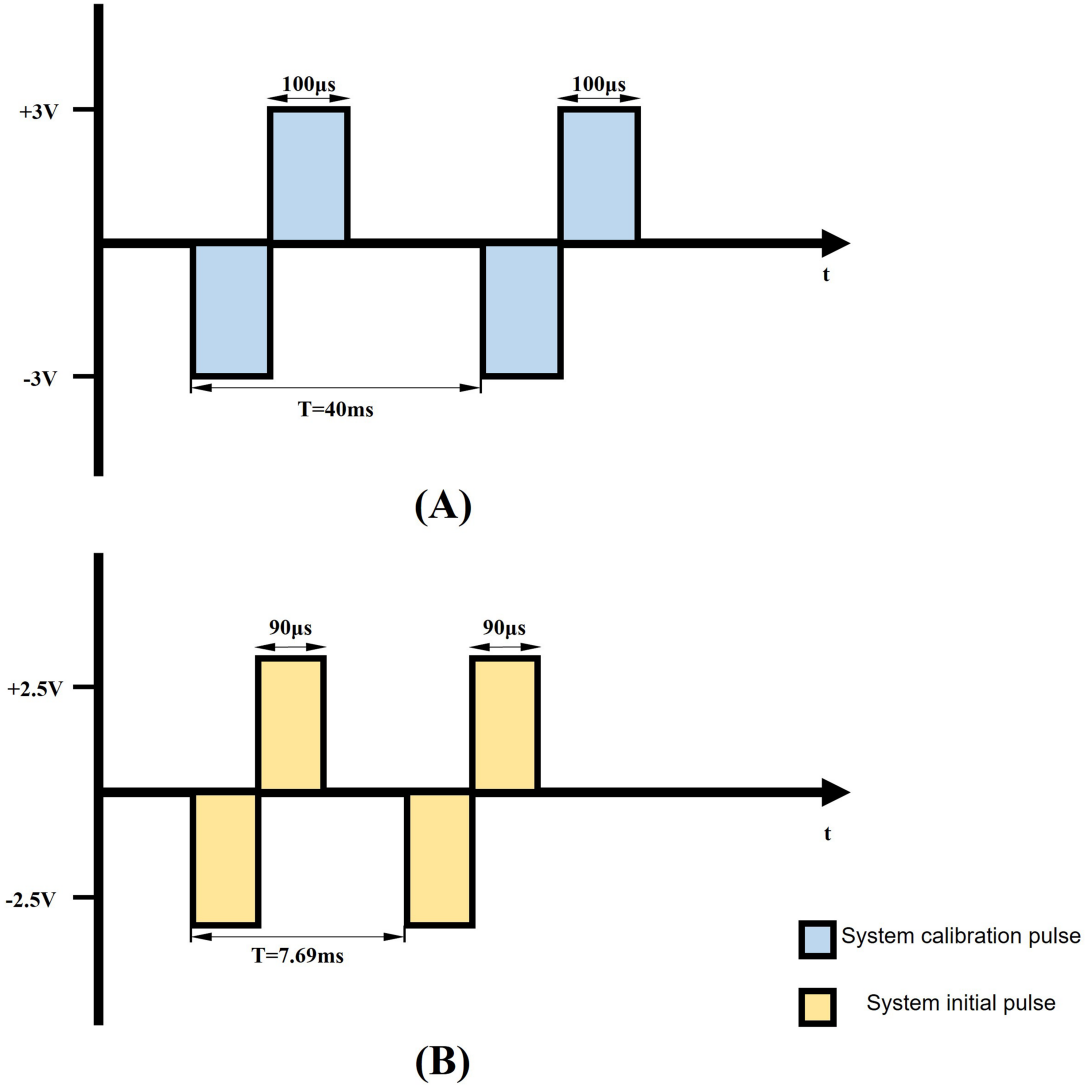
Schematic representation of biphasic pulse waveform. **(A)** The schematic depicts a 200 μs pulse width, 25 Hz biphasic constant-voltage pulse (±3 V), used for DBS pulse calibration. **(B)** The diagram illustrates the system’s initial output parameters: 130 Hz biphasic constant-voltage pulses (±2.5 V) with 180 μs pulse width.

The microstimulator delivered biphasic square-wave pulses with programmable parameters: frequency (80–150 Hz, 10 Hz increments), pulse width (40–340 μs, 50 μs steps), and amplitude (2.3–3 V, 0.05 V resolution). The corresponding control algorithms for these parameters were implemented in the host computer software. The biphasic pulse waveform characteristics are graphically depicted in Figure 5. Between consecutive pulses, the output voltage maintains zero potential (Figure 5A), preventing charge accumulation in the surrounding implanted tissue.

To account for inter-subject variability, stimulation parameters were individually calibrated for each rat by gradually increasing the voltage amplitude from an initial 2.5 V, with incremental adjustments of 0.05 V, while monitoring behavioral responses. Parkinsonian rats exhibited a characteristic progression, starting with contralateral orofacial twitches and progressing to severe forelimb motor dysfunction as the stimulation intensity increased from baseline settings (130 Hz frequency, 90 μs pulse width). The stimulation threshold was defined as the minimum voltage amplitude that elicited observable contralateral facial twitching. All subsequent stimulations were conducted at 0.05 V below this threshold to ensure subthreshold activation and avoid overt motor impairment.

## 3 Methods

### 3.1 Establishment of animal model

Rats were anesthetized with isoflurane (Sigma-Aldrich, United States) inhalation in a closed anesthesia induction box. Complete anesthesia was confirmed by the absence of corneal and nociceptive reflexes. Ophthalmic erythromycin ointment was applied to both eyes to prevent corneal dehydration and infection (Gambino et al., 2014), and body temperature was maintained at 37 °C using a heating pad (Huang et al., 2023). The rat was then secured in a stereotaxic frame (Stoelting Co., United States) (Yang et al., 2015; Castagnola et al., 2016), and surgical coordinates were determined using a brain atlas to guide the subsequent left intracerebral injection and electrode implantation procedures. After shaving the fur between the eye and ear lines, the skin was excised and disinfected. The periosteum was cleared from the skull surface using a 7.7% hydrogen peroxide solution (Sigma-Aldrich, United States), followed by the creation of a small craniotomy using a dental drill.

**Figure 6:**
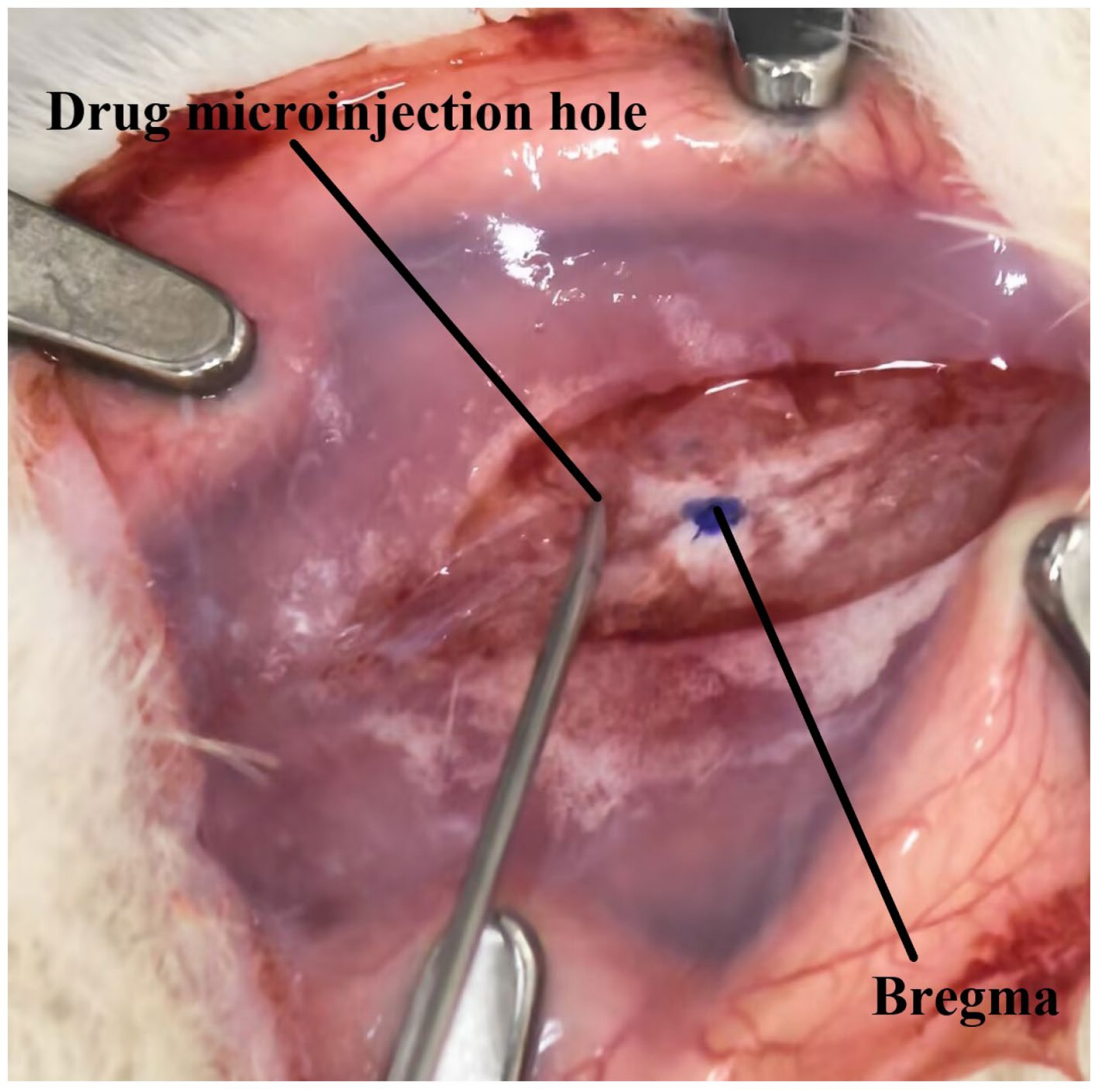
Schematic of the microinjection experimental rat model. Including the bregma and microinjection site.

A total of 12 rats were used in this study for PD model construction. Rats in the PD group were treated with 6-OHDA (4 μL, 5 μg/μL dissolved in 0.2% ascorbic acid and 0.9% normal saline) in a microsyringe. Purchased from Sigma-Aldrich, United States were injected slowly into the left MFB area at a rate of 1 μL/min (AP = −4.4 mm, ML = −1.2 mm, DV = −7.8 mm) to establish a unilateral PD model. In the sham experimental group, 0.2% ascorbic acid (4 μL, purchased from Sigma-Aldrich, United States) was slowly injected into the left MFB area (AP = −4.4 mm, ML = −1.2 mm, DV = −7.8 mm) with a microsyringe at a rate of 1 μL/min. Following surgery, meloxicam solution (purchased from China National Pharmaceutical Group Corporation) was administered to alleviate pain. One week after 6-OHDA lesioning, apomorphine (0.5 mg/kg, purchased from Sigma-Aldrich, United States) was administered subcutaneously to induce rotational behavior, which was used to assess the degree of dopamine depletion and evaluate the progression of the Parkinson’s disease model. When the net contralateral rotations exceeded 210 turns in 30 min, the model was considered successfully established (Wang et al., 2024). Data from rats that died during the experiment were excluded from the final statistical analysis. The schematic of the microinjection site is shown in Figure 6.

All experimental procedures were performed in accordance with the ARRIVE guidelines and approved by the Research Ethics Committee of Chongqing University of Posts and Telecommunications (Approval No. CSTBLLZM202402). Efforts were made to minimize both the number of animals used and the pain experienced during the experiments. Sprague–Dawley rats (male, weighing 180–260 g, purchased from the Southwest University Laboratory Animal Co., Ltd.) were housed in standard SPF animal rooms (Yang et al., 2021), with one rat per cage, at a temperature of 21 °C and a 12-h light/dark cycle. Food and water were provided ad libitum (Ahmed et al., 2010). After the experiment, all rats were euthanized with sodium pentobarbital solution (0.15 mg/g, purchased from Sigma-Aldrich, United States). The euthanasia procedure described above was carried out in strict accordance with the guidelines given by the AVMA.

### 3.2 Electrode parameters and electrode implantation

For this study, custom-designed micro-wire array electrodes were utilized to ensure long-term stimulation stability. The stimulation arrays were fabricated using nichrome alloy wires (35 μm diameter, 10 mm length, Plexon Inc., Beijing) arranged in a 1 × 3 matrix with 250 μm inter-electrode spacing. Each array incorporated two 125 μm-diameter silver wires at the connector interface, which served as reference electrodes. The active stimulation region featured a precisely controlled 3 mm exposed tip, while the remaining length was insulated with a 10 μm-thick polyimide layer (dielectric strength >200 V/μm) and encapsulated with polyethylene glycol (PEG-400) for mechanical stabilization and biocompatibility.

**Figure 7:**
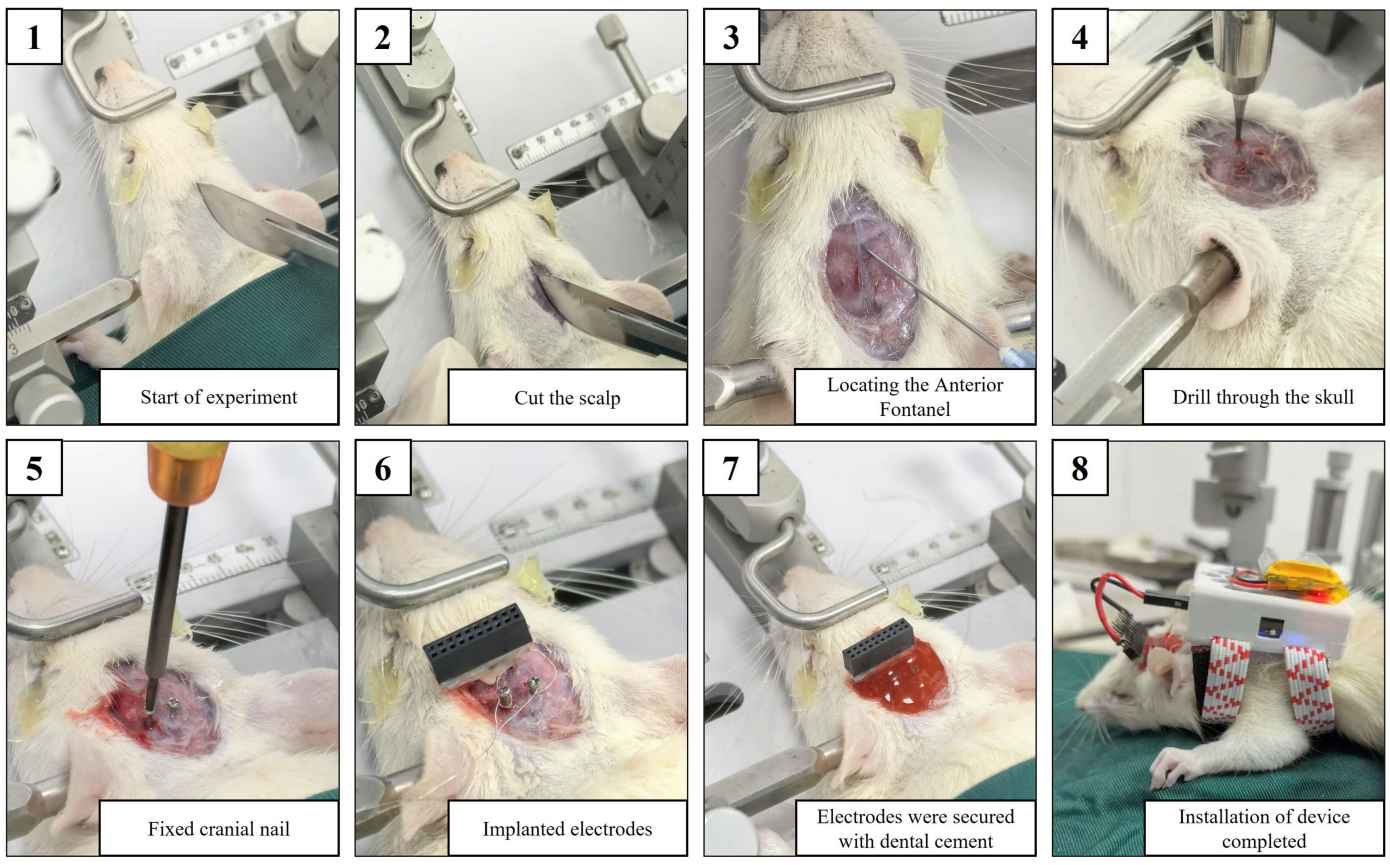
Implantation of electrodes and procedures of surgery are shown from 1 to 8 subsequently.

The stimulation electrodes were stereotactically implanted into the left subthalamic nucleus (STN) of PD rats (coordinates: AP = −4.4 mm, ML = −1.2 mm, DV = −7.8 mm relative to bregma). Four stainless steel screws (1 mm diameter) were surgically secured to the skull surface to provide anchoring points for the ground wires. The electrode assembly was permanently affixed to the skull using dental acrylic (Jet Denture Repair Acrylic, Lang Dental), with the screws serving as mechanical anchors. Postoperatively, rats received daily intraperitoneal inject-tions of penicillin G and meloxicam for three consecutive days to prevent infection and alleviate surgical pain. The detailed experimental procedure is shown in Figure 7.

### 3.3 *In vitro* verification and behavioral analysis

#### 3.3.1 Waveform output and neural models test

**Figure 8:**
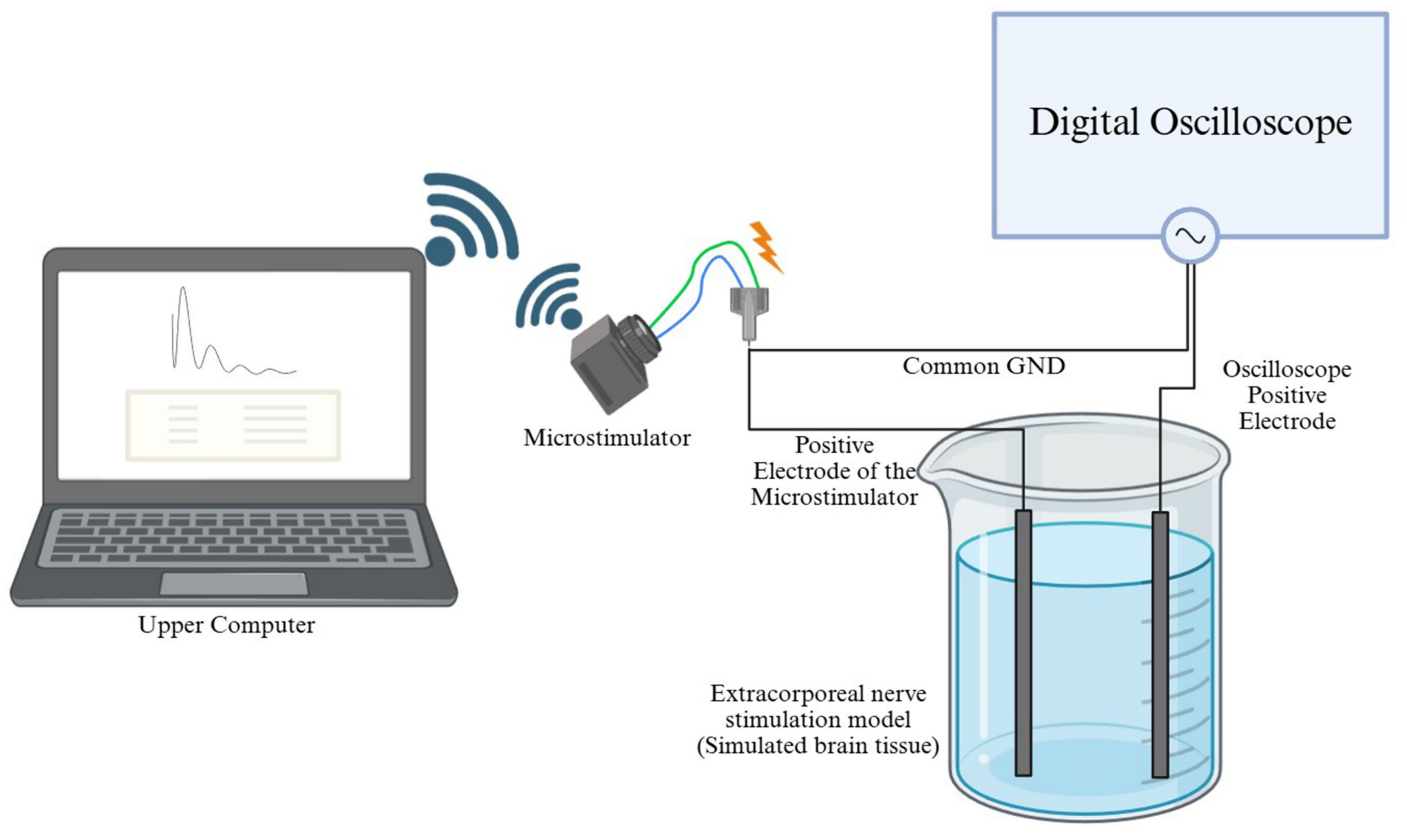
Schematic illustration of electrical stimulation in an *in vitro* model of nerve stimulation.

The response of the stimulator plays a crucial role in determining the efficacy of stimulation. To verify the accuracy and stability of the waveform, electrodes were immersed in a physiological saline gel (1% agar, 0.9% NaCl, 0.05% KCl), which mimicked the properties of biological tissue, thereby enabling quantitative assessment of signal attenuation rates. Waveform parameters, including frequency, pulse width, and amplitude, were precisely measured using a digital oscilloscope (Tektronix TBS1052B, United States) connected to the electrodes via probes. Strict environmental controls were maintained throughout all experiments. Figure 8 presents the schematic of the electrical stimulation setup in the *in vitro* neural stimulation model.

#### 3.3.2 Behavioral tests

To minimize circadian influences on rodent behavior, all behavioral tests were conducted within consistent time windows each week. Thirty minutes before testing, rats were acclimated to the experimental chamber in dedicated transfer cages to minimize stress-related artifacts. Between trials, the experimental apparatus was thoroughly cleaned using water and 75% alcohol to remove fecal residue and odor contamination from the previous subjects.

The open field apparatus consisted of a 75 cm × 75 cm acrylic base with a 40 cm high transparent acrylic enclosure. Testing took place in a quiet room with moderate lighting. After placing the rat in the center of the device, behavioral trajectories were recorded using an overhead high-definition camera (3D Motion Trajectory Capture System of NOKOV Series).

For the beam test, rats were tested for motor performance on a 50 × 1.2 cm (L × W) beam. The test beam was 30 cm from the table top, and a nylon hammock was installed 7.5 cm above the table top to provide cushions and mitigate the effects of falls on the experimental animals. A light source was placed at the starting end as an aversive stimulus to drive the experimental animal through the beam. When the experimental animal falls in the experiment, it needs to put the animal back to the starting point and start the test again until the animal reaches the other end of the beam, and the test time and the number of falls are finished.

For the cylinder test, animals were placed in a 15 × 20 cm (D × H) cylinder for 15 min. The number of contacts between the upper limbs on different sides of the cylinder wall in the upright state was tested and recorded. After obtaining the relevant experimental data, the correlation index was obtained using the calculation formula.

**Figure 9:**
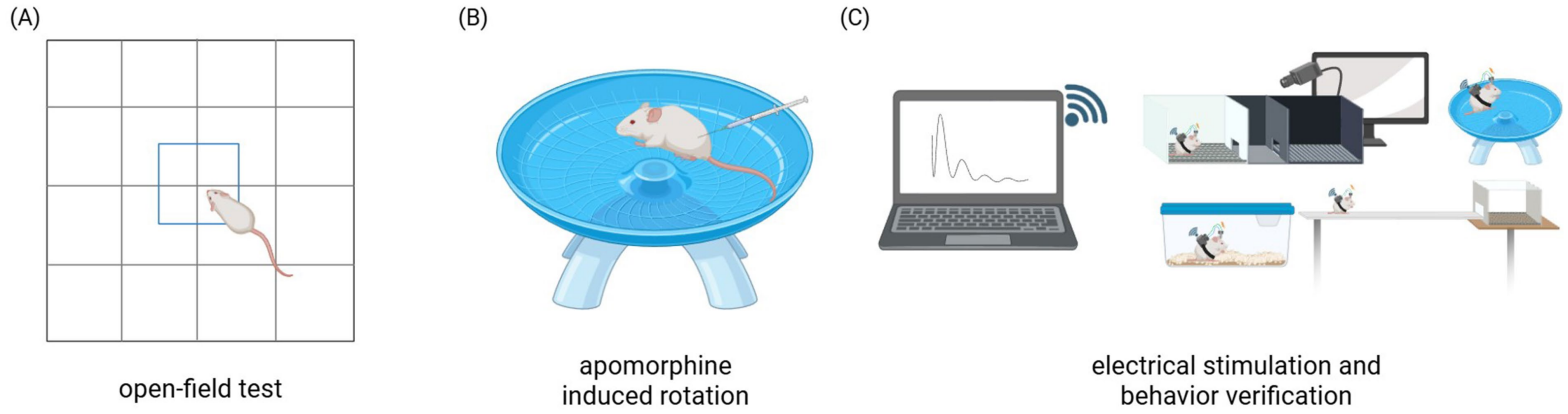
Schematic representation of the behavioral test. **(A)** Schematic diagram of the open-field test. **(B)** Schematic diagram of apomorphine-induced rotation test. **(C)** Schematic representation of PD-DBS rat groups under different behavioral tests.

To prevent learning effects from repeated testing, the open field test was performed only once per week, ensuring that the behavioral data reflected true individual differences. A schematic of the test procedure is shown in Figure 9. All data were recorded and archived simultaneously by two independent observers using the high-definition camera (3D Motion Trajectory Capture System of NOKOV Series).

### 3.4 Statistical analysis

All statistical analyses were performed in SPSS 21.0 software (SPSS, Inc., Chicago, IL, United States). After screening the data for completeness and outliers, the Shapiro–Wilk test was used to assess the normality of the data. All statistical tests obtained in the final analysis took *p* < 0.05 as the criterion for statistically significant difference to ensure the rigor and credibility of the comparison results between groups.

## 4 Results

This study presents the preliminary design and validation of the device. To ensure its effective application in rodent PD models, a comprehensive, multidimensional evaluation was conducted, with a primary focus on the following two aspects.

### 4.1 Waveform output test

**Figure 10:**
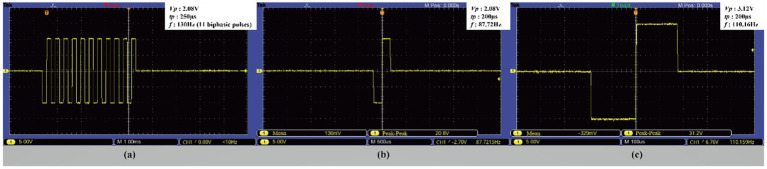
Stimulus signal output. **(a)** During a stimulating pulse cycle, the pulse amplitude was 2.08 V, the pulse width was 250 μs, the frequency was 130 Hz, and each group of pulses consisted of 11 bipolar pulses. **(b)** During a stimulating pulse cycle, the pulse amplitude was 2.08 V, the pulse width was 200 μs, and the frequency was 87.72 Hz. **(c)** During a stimulating pulse cycle, the pulse amplitude was 3.12 V, the pulse width was 200 μs, and the frequency was 110.16 Hz.

Given that the electrical conductivity of the constructed *in vitro* neural stimulation model closely approximates that of biological brain tissue, this study systematically evaluated the attenuation rates of two DBS output waveforms under both unloaded and gel conditions. The results demonstrate that signal attenuation in both environments was less than 8%, satisfying the predefined experimental criteria. Since the oscilloscope probe was set to ×1, the actual measured amplitude is one-tenth of the displayed value. Figure 10 presents the output of the stimulation signal under different parameter settings. In Figure 10A, the stimulation pulse cycle has a frequency of 130 Hz, with each pulse train consisting of 11 biphasic pulses (amplitude 2.08 V, pulse width 250 μs). Figure 10B displays a stimulation pulse cycle composed of a single pulse (amplitude 2.08 V, pulse width 200 μs, frequency 87.72 Hz). Figure 10C shows an adjusted stimulation pulse cycle with a single pulse (amplitude 3.12 V, pulse width 200 μs, frequency 110.16 Hz).

### 4.2 Animal behavioral experiments

**Figure 11:**
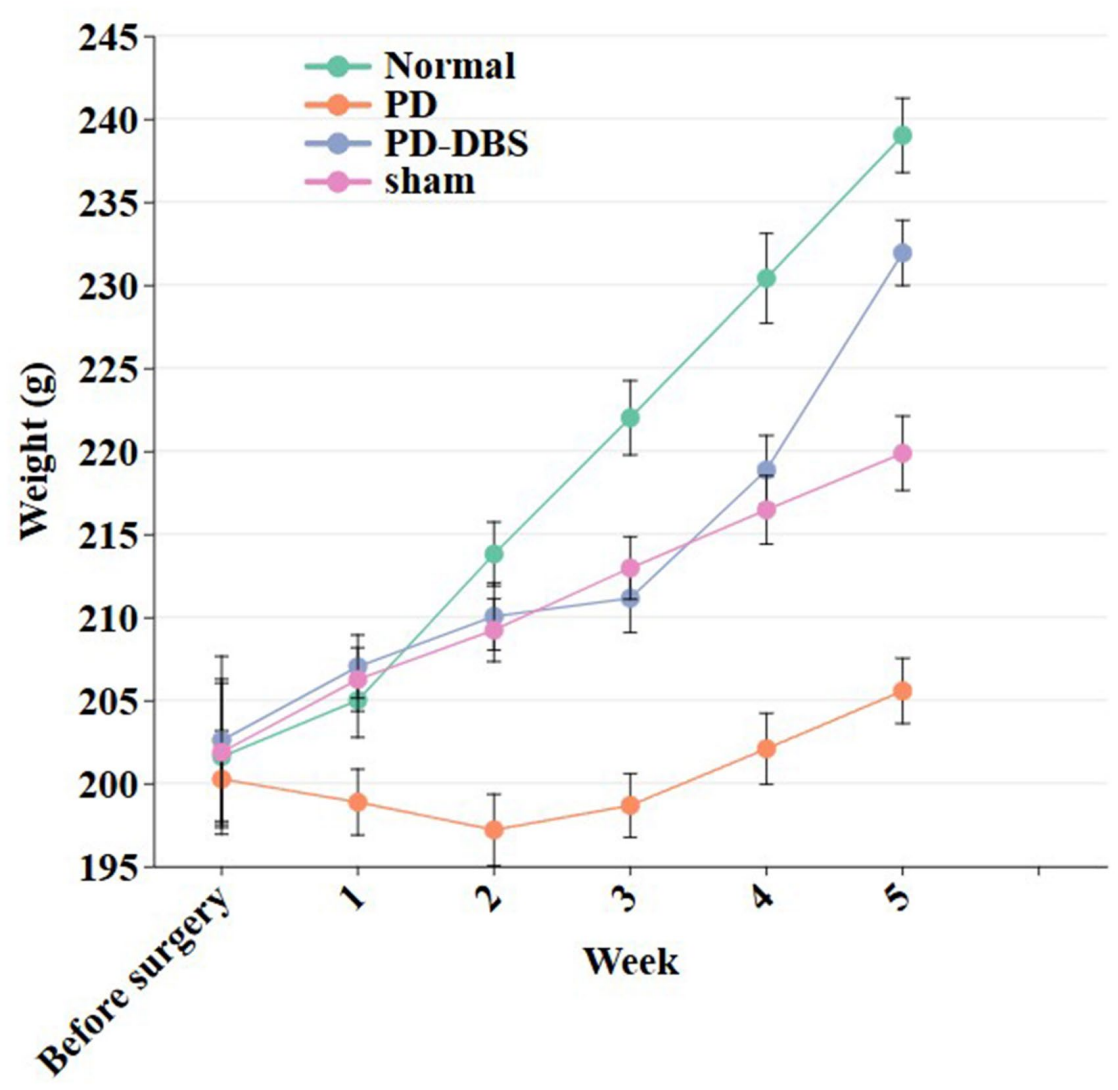
Weight change of rats. In the PD-DBS group, the stimulation frequency was 80 Hz during weeks 3–4, and 130 Hz during weeks 4–5.

To test the compatibility of the implanted device, weight monitoring was performed on all experimental animals. The rats in the surgical group experienced a maximum weight loss of 3.5% of their initial body weight post-surgery, which is within the generally accepted standard for stress-free surgery. All animals showed normal foraging and drinking habits after operation, that is, when food was placed and drinking water was changed at a fixed time every day, there was no significant difference in food intake and drinking water intake between each caged animal and normal rats, and there was no reduction in food intake and drinking water caused by postoperative pain and other factors. In addition, in the familiar environment of the cage, the activity state of the sham operation group was consistent with that of the normal rats, and the vitality was normal. The rats in the PD model group showed a slight decrease in activity due to the symptoms related to the disease model, but did not show abnormal behavior. In conclusion, no pain-related manifestations were observed in all animals after surgery and their clinical condition was stable. The weight changes of rats in all groups are shown in Figure 11.

To evaluate the behavioral performance and spontaneous locomotor activity of rats, key indicators such as total movement distance, time spent in the central area, and bilateral limb balance were compared across experimental groups. Compared to the stimulation control group, PD rats exhibited markedly decreased locomotor activity and reduced central zone exploration, reflecting typical behavioral impairments associated with Parkinsonian models. In contrast, animals subjected to 1 week of electrical stimulation showed significantly enhanced exploratory behavior. These rats traveled longer distances and spent more time in the center area than those in the PD and sham groups during the same observation window. Notably, rats receiving 130 Hz stimulation spent more time in the central zone than those in the 80 Hz group, indicating a frequency-dependent behavioral modulation effect.

**Figure 12:**
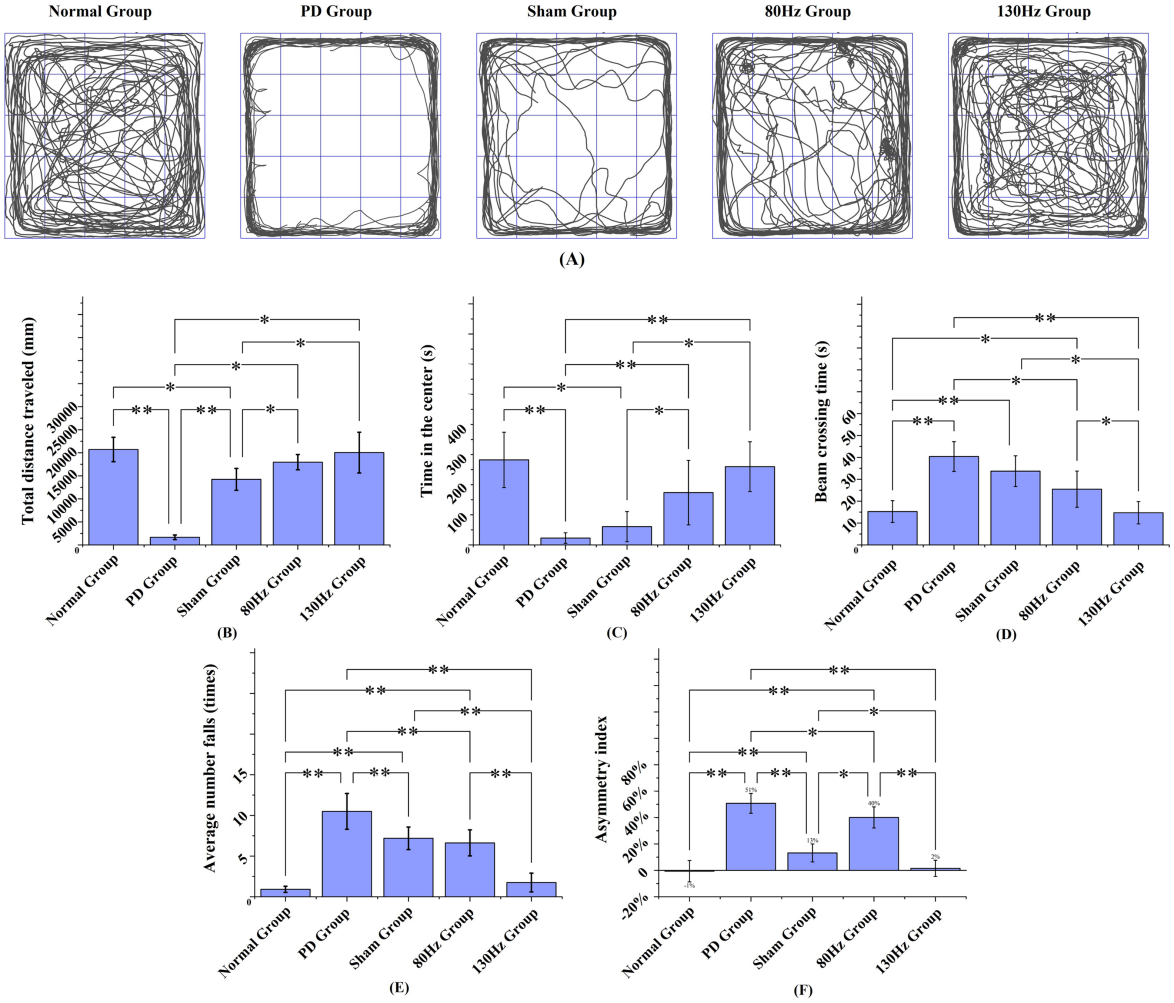
Behavioral performance analysis across experimental groups. **(A)** Representative locomotor trajectories during three 15 min open-field sessions, illustrating group-wise differences in spatial exploration. **(B)** Total distance traveled in the open-field test. **(C)** Duration spent in the central area during the open-field test. **(D)** Beam-crossing time in the beam-walking test as a measure of trunk motor coordination. **(E)** Number of falls during beam walking. **(F)** Forelimb asymmetry index obtained from the cylinder test. * *p* < 0.05, ** *p* < 0.01, *** *p* < 0.001.

In the open-field test, Figure 12A illustrates the spatial exploration patterns of all groups. Figure 12B shows that rats in the 130 Hz stimulation group achieved nearly normal locomotion distances, significantly outperforming the PD and sham groups. Figure 12C confirms that both stimulation groups spent more time in the central region compared to the PD group. In the motor coordination assessment, the beam walking test revealed that PD rats required longer traversal times and made more frequent slips, whereas stimulation intervention effectively mitigated these motor impairments, as shown in Figures 12D,E. Additionally, the forelimb asymmetry index, derived from the cylinder test, demonstrated significant improvement in the stimulation groups. Notably, rats in the 130 Hz stimulation group exhibited forelimb usage symmetry comparable to that of healthy controls, as illustrated in Figure 12F.

Taken together, these results suggest that the proposed stimulation system effectively alleviates motor deficits in PD rats and provides adaptable stimulation parameters to meet diverse experimental needs. The observed outcomes align with the intended functional design of the device.

## 5 Discussion

In the study, our device significantly surpasses existing designs in terms of usability, Wearability, and flexibility. The battery life of our device notably exceeds that of the low-cost, backpack-style multichannel DBS system proposed by Tala et al. (2021). Moreover, compared with the systems by Grotemeyer et al. (2024) and Fleischer et al. (2020), the independent power supply structure incorporated into our design greatly enhances the ease of battery replacement without interrupting stimulation sessions. Additionally, our simplified and highly integrated circuit design reduces the total device cost to below $18, providing an advantageous price-performance ratio relative to previous devices by Tala et al. (2021) and Alam et al. (2013).

Crucially, the behavioral outcomes from our hemi-PD rat model provide compelling evidence for the functional efficacy of our stimulator. The data presented in Figure 12 allow us to draw meaningful inferences regarding the relationship between stimulation parameters and behavioral recovery. The wearable DBS device proposed in this study can effectively improve the behavioral deficits of PD models through chronic and adjustable stimulation, and its stimulation effect is obviously consistent with the stimulation mentioned in the existing research on the mechanism of DBS, that is, high-frequency stimulation is more effective in improving the abnormal tremor caused by PD (Baker et al., 2023).

Our results indicate a clear frequency-dependent effect on motor and exploratory behavior. Rats receiving 130 Hz stimulation demonstrated superior performance compared to both the 80 Hz group and the unstimulated PD groups. Specifically, as quantified in Figures 12B,C, the 130 Hz group not only recovered near-normal locomotion distances but also exhibited a significant increase in the time spent in the anxiogenic central zone of the open field. This latter finding is particularly insightful, as it suggests that effective STN-DBS may not only ameliorate bradykinesia but also mitigate the anxiety-like behaviors often comorbid with PD, potentially by restoring normal exploratory drive.

Significant and consistent behavioral deficits were observed in the unstimulated PD group during postoperative recovery and behavioral testing. These deficits included a markedly reduced locomotor activity in the open field test (Figures 12B,C), poor trunk coordination as reflected by significantly longer beam-crossing times and more frequent slips (Figures 12D,E), and a persistent forelimb asymmetry in the cylinder test (Figure 12F). The animals in the stimulation group showed significantly improved behavior. This indicates that the stimuli provided by the stimulator in this study were effective. This stark contrast highlights the necessity of DBS intervention for functional recovery of this model.

In addition, the beam walking test results provide a relatively effective Angle to observe the motion recovery. The absence of improvement over time in the PD group and the significantly shorter crossing times in the stimulation group (specifically in the 130hz group) suggest that continuous DBS provides acute motor facilitation, which may contribute to the execution of complex coordinated motor skills, creating a state of tolerance for normal motor planning and execution. As shown in Figure 12F, the asymmetry index of the forelimbs in the 130 Hz group significantly improved and approached the normal level, indicating an improvement in bilateral movement balance, further confirming this view.

In summary, the behavioral analysis confirms that our wearable DBS system is not merely a functional stimulator but a refined research tool capable of eliciting parameter-specific, therapeutically relevant improvements in a PD model. The ability to chronically deliver and wirelessly adjust these parameters was instrumental in uncovering these frequency-dependent effects, thereby validating the device’s design goals of flexibility, reliability, and efficacy for long-term behavioral studies.

Additionally, although our device provides an extended operational time exceeding 30 days, long-term studies may require even more robust battery solutions or wireless energy transfer technologies to further minimize operational disruptions (Forni et al., 2012; Fleischer et al., 2020; Grotemeyer et al., 2024). Integration of advanced energy-efficient components, such as ultra-low-power microprocessors and optimized Bluetooth communication protocols, may also extend the device’s battery life and enhance its reliability in long-term studies (Alam et al., 2013; Even-Chen et al., 2020).

Compared to other recent developments, including the software-defined implantable modular platform (STELLA) proposed by Plocksties et al. (2021), and the implantable microprocessor integrating two-photon imaging technology proposed by Alpaugh et al. (2019), our system prioritizes flexibility and ease of use. However, integrating elements from these systems, such as enhanced programmability post-implantation and multi-modal sensing capabilities, could further improve device functionality and broaden research applications. Additionally, exploring emerging technologies such as wireless, battery-free stimulation devices demonstrated by Burton et al. (2021) could inspire future innovations aimed at completely eliminating the limitations imposed by battery life.

In summary, while our device substantially advances current rodent DBS research, continued technological improvements—especially integrating adaptive feedback, optimizing energy management, and expanding functional capabilities—are critical for future developments. These refinements will further facilitate comprehensive and precise neuroscientific investigations into the mechanisms underlying DBS treatments and neurological disorders.

## 6 Conclusion

In this study, we developed a wearable DBS device for rodent behavioral research, addressing limitations of existing systems. The device features lightweight design, extended battery life, and adjustable stimulation parameters (frequency: 80–150 Hz, pulse width: 40–340 μs, amplitude: 2.3–3 V). Behavioral experiments in hemi-PD rat models confirmed its effectiveness in alleviating motor deficits. Although our DBS device offers significant advantages, improvements are still needed. Current limitations include the lack of bio-signal acquisition for adaptive closed-loop stimulation. Therefore, in our future work, we can continue to focus on two main directions. On the one hand, it focuses on expanding the applicable scope of stimulation parameters of the device. On the basis of the existing optimized Settings for Parkinson’s disease, it further supports a wider range of parameters including lower frequencies, so as to adapt to the treatment needs of various neuropsychiatric diseases such as dystonia and epilepsy, and improve the clinical versatility and treatment customization ability of the device. On the other hand, the system function is upgraded from “stimulation only” to “stimulus and acquisition integration.” By integrating real-time neural signal recording and stimulus artifact elimination technology, multi-target and multi-brain region EEG signals and behavioral data are collected synchronously, so as to reveal the mechanism of deep brain stimulation and its intrinsic relationship with Parkinson’s disease treatment at the dual level of loop and behavior.

## Data Availability

The original contributions presented in the study are included in the article/supplementary material, further inquiries can be directed to the corresponding author.
